# Exploring the protective role of DDIT4/mTOR in podocyte integrity through macrophage polarization in diabetic kidney disease

**DOI:** 10.1080/0886022X.2025.2546623

**Published:** 2025-09-07

**Authors:** Jingxuan Shi, Xiansen Wei, Guming Zou, Xinze Liu, Jiaqi An, Qiaoya He, Yuanyuan Jiao, Jingwei Tian, Yue Yang, Li Zhuo, Wenge Li

**Affiliations:** ^a^ Department of Nephrology, China-Japan Friendship Hospital, Beijing, China; ^b^ China-Japan Friendship Institute of Clinical Medical Sciences, Beijing, China; ^c^ Beijing University of Chinese Medicine China-Japan Friendship Clinic Medical College, Beijing, China; ^d^ China-Japan Friendship Clinic Medical College, Peking University, Beijing, China; ^e^ Department of Nephrology, Fuwai Hospital, Chinese Academy of Medical Science, Beijing, China; ^f^ Department of Nephrology, Beijing Sixth Hospital, Beijing, China

**Keywords:** Diabetic kidney disease, DDIT4, mTOR, macrophages, inflammation, podocytes

## Abstract

**Objectives:**

In this study, we explored the mechanism by which DDIT4 influences the polarization phenotypic transformation of macrophages and inflammation through the regulation of mTOR signaling pathway, providing a new mechanism and target for the treatment of diabetic nephropathy.

**Methods:**

The degree of inflammation and injury in renal tissues of diabetic kidney disease (DKD) animal model was evaluated using biochemical assays, renal pathology examinations, and Western blot tests. Podocytes and macrophages were isolated from renal tissues to observe the extent of podocyte injury and the quantity and polarization phenotype of macrophage infiltration. Subsequently, the activated M1 macrophage model was constructed and transfected with DDIT4 to evaluate the effect of DDIT4 on the polarization phenotype transformation of macrophages and the expression of inflammatory factors. Finally, the co-culture system of macrophages and podocytes was constructed to detect the cell apoptosis, and the morphology and subcellular structure of podocin were observed by transmission electron microscopy.

**Results:**

In the DKD animal model, the expression levels of inflammatory factors in renal tissues were significantly higher than those in the control group. Additionally, there was significant damage to the renal podocytes. At the same time, there was a higher number of macrophage infiltrations, predominantly of the M1 polarized phenotype. In the constructed M1 polarized macrophage model, overexpression of DDIT4 can induce the decrease of M1 macrophages and reduce the expression level of inflammatory factors. In the co-culture system of macrophages and podocytes, overexpressed DDIT4 significantly reduced the proportion of podocyte apoptosis and protect the changes of morphology and subcellular structure.

**Conclusions:**

The ability of DDIT4 to mediate the transformation of macrophage phenotype and reduce inflammation reveals its potential as an innovative drug discovery target for DKD. Further exploration and validation of the therapeutic potential of DDIT4 may provide effective interventions to address unaddressed clinical needs in the treatment of DKD.

## 1. Introduction

Diabetes mellitus (DM) is one of the fastest growing diseases worldwide, with 693 million adults expected to have diabetes by 2045 [1], and diabetic kidney disease (DKD) is one of the most common complications of diabetes and the leading cause of end-stage kidney disease (ESRD). The pathogenesis of DKD is multifaceted and, involves renal hemodynamic alterations, inflammatory responses, metabolic dysregulation, and oxidative stress [2]. A key characteristic of DKD is systemic and localized kidney inflammation, which leads to renal fibrosis and structural remodeling [3,4]. Inflammation is generally believed to play an important role in the occurrence and development of DKD [5], and macrophages are considered the key cells mediating kidney inflammation and promoting DKD development [6]. According to the different activation modes and functions, macrophages are classified into the classically activated M1 phenotype and substitutively activated M2 phenotype. M1-type macrophages are mainly involved in the pro-inflammatory response, while M2-type macrophages are mainly involved in anti-inflammatory responses [7]. M1-type macrophages produces a large number of inflammatory mediators through the release of tumor necrosis factor-α (TNF-α), interleukin-1β (IL-1 β), interleukin-6 (IL6) and other pro-inflammatory cytokines. Triggering the inflammatory response and mediating tissue damage [8]. M2-type macrophages secrete anti-inflammatory cytokines such as interleukin-10 (IL-10) and interleukin-4 (IL-4), which participate in the repair stage of injury, inhibit the inflammatory response, and promote tissue repair, remodeling, and healing [9,10]. Previous studies have revealed that during the progression of DKD, the proportion of M1-type macrophages infiltrating in the kidney increases significantly, whereas the proportion of M2-type macrophages decreases [11,12]. In addition, in the kidney of the DKD rat model, it was found that the expression of M1-type macrophage markers CD80 and CD86 increased, while the expressions of M2-type macrophage markers CD163 and CD206 decreased, indicating that the transformation of macrophages to the M1 phenotype played an important role in promoting the progression of DKD [13]. In recent years, the interaction between macrophages and podocytes in DKD has attracted considerable attention. Podocytes play an important role in the construction and function of the glomerular filtration barrier [14]. Apoptosis, caused by sustained injury of podocytes, is considered a central event in the occurrence and development of DKD [15]. Macrophage-mediated podocyte apoptosis is an important component of DKD [16]. M1-type macrophages play a pro-inflammatory role and destroy the integrity of podocytes’ structure and function by secreting TNF-α and other key factors related to the development of inflammation, which directly leads to early DKD kidney injury [17]. Our previous studies have shown that DDIT4 enhances autophagy and reduces oxidative stress by regulating the VDR-mTOR pathway, and alleviating the occurrence and development of DKD. This indicated that DDIT4 plays a positive role in the treatment of DKD [18]. DDIT4, also known as Developmental and DNA Damage Response Regulation 1 (REDD1) or Di92, is located on the human chromosome 10. It can be increased in response to hypoxia, oxidative stress, DNA damage, and other stressors, affecting processes such as apoptosis and energy stress [19,20]. In a recent study, increased DDIT4 expression was shown to induce downregulation of pro-inflammatory cytokines, such as nuclear factor κB (NF-κB) and markers related to oxidative stress. Moreover, this process is related to macrophage regulation of macrophages [21]. Recent evidence suggests that DDIT4-mTOR signaling mediates macrophage polarization in diabetic complications [22]. However, whether this axis governs renal macrophage phenotypic switching in DKD remains unexplored. Therefore, this study hypothesized that the DDIT4-mTOR pathway is involved in regulating the ratio between the M1 and M2 phenotypes of macrophages, inhibiting the expression of inflammation-related factors, reducing the apoptosis of podocytes, and playing a role in the treatment of DKD. To test this hypothesis, we used db/db mice as a DKD model and m/m genotype litters as blank controls to evaluate renal tissue injury, macrophage infiltration, and inflammatory cytokine expression in DKD animal models. Subsequently, we constructed an activated macrophage model and validated the DDIT4 transfection. After confirming successful DDIT4 transfection, we used immunofluorescence, flow cytometry, and western blotting to analyze their influence on the phenotypic transformation of macrophages, and further explored the changes in the damage effect of macrophages on podocytes after the intervention. It was confirmed that the DDIT4-mTOR signaling pathway affects the development of inflammation by regulating the polarization phenotype of macrophages, and plays a protective role in podocytes.

## 2. Materials and methods

### 2.1. Experimental animals

Six-week-old male SPF db/db mice and their littermate controls were obtained from Hangzhou Ziyuan Laboratory Animal Technology Co., Ltd. Mice were fed a high-fat diet under SPF conditions to induce a DKD-associated phenotype. The weight, eating habits, mental state, coat color and activity level of each mouse group were recorded weekly. Each group of mice (control group *n* = 3, model group *n* = 3) underwent 24-h urine collection using metabolic cages after 10 weeks of intervention, followed by random blood glucose testing *via* tail vein blood sampling. Terminal anesthesia was administered before whole blood collection through cardiac puncture, and mice with random blood glucose >16.7 mmol/L were included in the model group. The creatinine and urine protein values were detected according to the manufacturer’s instructions (Solarbio, Beijing, China). Kidney tissues were collected for further pathological analyses. Renal tissue was collected from live laboratory animals under anesthesia. All procedures were conducted in compliance with ethical guidelines and approved by the relevant ethics committee. Furthermore, we implemented a humane approach in the experimental design to ensure minimal distress and discomfort throughout the process. The collection procedure was performed in accordance with ethical guidelines and strict aseptic conditions to ensure the integrity and quality of the samples for subsequent experimental testing. Additionally, all animal experiments were approved by the Animal Ethics Committee of China-Japan Friendship Hospital (No. zryhyy61-23-02-11) and conducted in accordance with the ARRIVE guidelines.

### 2.2. Podocyte isolation from kidney tissue

Mouse kidney tissues were screened using the differential screening method, and glomeruli with 300 meshes were obtained. The prepared KI-3T3 was used to culture glomeruli and then placed in a Petri dish covered with collagen from rat tails. After four days of culture, the medium was changed. After seven days, trypsin was used to digest the glomeruli for passaging and culturing of podocytes. Passaging was performed every five to seven days. After two to three passages, the morphology of podocytes was observed and collected for subsequent experiments.

### 2.3. Periodic acid Schiff staining (PAS)

Mouse kidney tissue was fixed in 4% paraformaldehyde, embedded, and sectioned at 4 μm thickness. The slices were dewaxed with xylene, hydrated with a gradient of ethanol, stained with alcian blue dye for 10–20 min, oxidized with 1% periodate for 5 min, stained with Schiff dye for 10–20 min, counterstained with hematoxylin dye for 2 min, differentiated with a 1% hydrochloric acid ethanol solution for 2–3 s, turned blue with ammonia for 3 min, dehydrated with gradient ethanol, and made transparent with dimethylbenzene. After sealing with neutral gum, slices were examined and photographed under a microscope (IX71, OLYMPUS).

### 2.4. Immunohistochemical staining (IHC)

Mouse kidney tissues were fixed with 4% paraformaldehyde, and after paraffin embedding, continuous sectioning was performed at a thickness of 4 μM. The slices were rehydrated with gradient ethanol solution, repaired with citrate antigen repair solution at 95 °C for 15 min, inactivated with H_2_O_2_ at room temperature for 10 min, permeabilized with 0.1% Triton X-100 at room temperature for 10 min, and blocked with a 5% BSA sealing solution at 37 °C for 1 h. Subsequently, we applied primary antibodies (COX2, Cxcl15, TNF-α, NF-κB和p-NF-κB) dropwise and incubated them at 37 °C for 2 h. The corresponding secondary antibodies were then added dropwise and incubated at room temperature for 20 min, after which DAB chromogenic agent was added for 5–10 min, the reaction was terminated using hydrogen peroxide and hematoxylin was used for nuclear staining for 1 min. Gradient ethanol dehydration, xylene transparent, drop neutral gum film, the stained slices were observed under a microscope and photographed.

### 2.5. Cell culture and differentiation induction

The mouse mononuclear macrophage leukemia cell line (Raw264.7) was cultured in Dulbecco’s modified DMEM medium (DMEM) containing 10% fetal bovine serum, 1% penicillin and streptomycin, and placed in an incubator at 37 °C and 5% CO_2_. When the cells were in the logarithmic phase, they were cultured in plates at a certain proportion. When the cells were in a stable state, the cell suspension was collected and, suspended in complete culture, and the cell concentration was adjusted to 1 × 10^5^ cells/mL, and inoculated in a 24-well culture plate. Phorbol-12-myristate-13-acetate (PMA) (Solarbio, Beijing, China) at a concentration of 25 ng/mL and Lipopolysaccharide (LPS) (Solarbio, Beijing, China) at a concentration of 300 ng/mL were added to each well, and cultured in an incubator at 37 °C and 5% CO_2_ for 24h and 48h respectively for subsequent experiments.

### 2.6. Cell transfection

Macrophages that were successfully induced and activated were collected for experiments. The cells were divided into three groups: A: activated macrophages; B: Activated macrophages + no-load group; and C: Activated macrophages + DDIT4 overexpression group. A clean aseptic centrifuge tube was taken, 25 μL DMEM medium without antibiotics and serum was added, 500 ng unloaded or DDIT4 overexpression vector was added, and then 0.8 μL Lipo8000TM transfection reagent was added (Beyotime, Shanghai, China). The cells were cultured at 37 °C for 4–6 h with a prepared mixed suspension and cell status was observed.

### 2.7. Flow cytometric differentiation detection

For *in vivo* analysis, mouse kidney tissue was placed in PBS containing a double antibody and rinsed three times. Ophthalmic scissors were used to cut the kidney into pieces, filter it with a sieve, collect the filtered cell suspension, and add lysis solution to lyse red blood cells. The cell pellet was resuspended in DMEM high-glucose basal medium (Gibco) and inoculated in a T25 square bottle for 2 h. The cells were gently washed with fresh medium, and adherent macrophages were collected for culture. The cultured macrophages were divided into 6 tubes, and each tube was added with 300 μL of Staining Buffer was, added to each tube. A certain proportion of CD11b + F4/80, rabbit anti CD86 and rabbit anti CD206 were added, and a negative control was set. The mixture was then incubated in the dark for 30 min. The cells were washed twice with 1 ∗ Perm/Wash buffer, resuspended in Staining Buffer, and analyzed by flow cytometry (FACS Verse, Becton, Dickinson Company). For the *in vitro* analysis of macrophage differentiation, the cultured and intervened macrophages were collected, a certain proportion of rabbit anti CD86 and rabbit anti CD206 were added, and a negative control was set. This method was repeated to detect the macrophage differentiation

### 2.8. Immunofluorescence staining (IF)

For the *in vivo* experiment, 4% paraformaldehyde was used to fix the kidney tissue, which was embedded in paraffin and sliced continuously at a thickness of 4 μM. The slices were rehydrated with a gradient ethanol solution, repaired with citric acid antigen repair solution at 95°C for 15 min, inactivated with H_2_O_2_ for 10 min at room temperature, permeabilized with 0.1% TritonX-100 for 10 min at room temperature, and sealed with 5% BSA sealing solution at 37°C for 1 h. The sections were incubated with primary antibody (CD86, CD206, F4/80) at room temperature for 2 h, then the corresponding fluorescent secondary antibody was added dropwise, incubated at room temperature for 1 h, Hochest was added at room temperature for 15 min, dehydrated with gradient ethanol, and sealed with anti-fluorescence quenching. The sections were observed and photographed under a fluorescence microscope (IX71, OLYMPUS). For the *in vitro* experiments, the cells were fixed with 4% paraformaldehyde for 15 min. PBS were washed three times with PBS and incubated with 5% FBS at 37°C for 2 h. The primary antibody (CD86, CD206, F4/80) was dropped, incubated overnight at 4 °C, then the secondary antibody corresponding to the primary antibody was added, and incubated for 1 h at 37 °C in the dark. Hoechst was incubated in the dark at room temperature for 15 min and then observed and photographed.

### 2.9. Western blotting (WB)

The tissue samples and cells were ground, mixed, and split with RIPA lysis buffer containing 1% PMSF (Beyotime, Shanghai, China) to extract the total protein. Protein concentration was determined using a BCA kit. The loading mass of each pore protein was 30 μg. Sodium dodecyl sulfate (SDS) polyacrylamide gel electrophoresis (PAGE) was used for electrophoresis, and the protein was transferred to a PVDF membrane. After 5% BSA was sealed at room temperature for 1 h, rabbit anti DDIT4 (1:2,000), rabbit anti Cox2 (1:1,000), rabbit anti Cxcl15 (1:1,000), rabbit anti TNF-α (1:1,000), rabbit anti NF-кB (1:1,000), rabbit anti P-NF-кB (1:1,000), rabbit anti- mTOR (1:1,000), rabbit anti p−mTOR(1:1,000), rabbit anti Nephrin (1:1,000) and rabbit anti podocin (1:1,000) were added. After overnight incubation at 4 °C overnight, the secondary antibody corresponding to the primary antibody was added and incubated at room temperature for 2 h. The ECL luminous solution develops color and collects images. GAPDH was used as an internal control.

### 2.10. Reverse transcription-polymerase chain reaction (RT-PCR)

The cells were adjusted to a density of 2 × 10^5^ cells/dish and inoculated in a 12-well plate. Each group of cells was treated with the corresponding amount of TRIzol lysate to lyse cells and extract total RNA from cells. After the total RNA was reverse transcribed into cDNA, it was amplified by real-time fluorescence quantitative PCR. The total PCR reaction system is 20 µL: SYBR Green Mix 10 µL, upstream primer 0.4 µL, downstream primer 0.4 µL, ddH2O 7.2 µL, cDNA template 2 µL. PCR reaction conditions: 94 °C pre denaturation for 10 min, 1 cycle; denatured at 94 °C for 20s, annealed at 55 °C for 20s, extended at 72 °C for 20s, 40 cycles in total.

### 2.11. Flow cytometry apoptosis detection

Cells cultured in the groups were collected by trypsin digestion without EDTA. 300 µL of 1*Binding Buffer suspension was added and mixed with 5 µL of Annexin V-FITC. The cells were incubated at room temperature for 15 min in the dark, followed by 10 µL of PI staining, and incubated at room temperature for 10 min in the dark. Flow cytometry was performed for detection and analysis.

### 2.12. Tunel staining

The cells cultured in the groups were collected and the culture medium in the cell sample pore plate was removed. The TUNEL working solution (Solarbio, Beijing, China) was added and incubated at 37 °C for 60 min. The TUNEL working liquid and wash one to two times with PBS. 4% paraformaldehyde at room temperature for 15 min. Hoechst stain was incubated for 10 min at room temperature in the dark. Observations were made using a fluorescence microscope, and photographs were taken and, analyzed by using ImageJ software.

### 2.13. Transmission electron microscopy (TEM)

Podocytes cultured in different media were collected and fixed with 2.5% glutaraldehyde for 3–4 h. The cells were centrifuged at 3,000 rpm at a low speed to collect the precipitate. After PBS cleaning twice with PBS, add 2.5% glutaraldehyde was added and fixed for 2 − 4 h. Cell samples were pre-embedded in 3% low melting point agar, rinsed with 0.1 M phosphoric acid rinse solution three times, and then placed in 1% osmic acid at 4 °C fixation for 2 h. The samples were successively dehydrated in 30%, 50%, 70%, 80%, 95% and 100% ethanol, and then 100% propylene oxide was added for 3 times. The embedded sample was penetrated with epoxy propane and the embedding solution at room temperature, and treated at 60°C for 48 h to fully polymerize the resin. An ultrathin microtome was used to slice at 70 nm, and 3% uranium acetate saturated ethanol solution was used for staining for 8 min, followed by staining with 2.7% lead citrate solution for 8 min. Images were observed and take photos under a transmission electron microscope (HT770-SS/FEITecnaiG20TWIN, HITACHI, Japan, FEI, USA).

### 2.14. Data analysis

The results are presented as the mean ± SD from at least three in dependent experiments using GraphPadPrism9 (La Jolla, CA, USA). Unpaired two- tailed Student’s t-test (two groups) and one-way ANOVA followed by Tukey’s *post hoc* test (≥ three groups) were used for continuous variables with a normal distribution and similar variances. Unpaired two-tailed Student’s t-test with Welch’s correction (two groups) and Brown-Forsythe and Welch ANOVA tests followed by Dunnett’s T3 multiple comparisons test (≥ three groups) were used for continuous variables with abnormal distribution and heterogeneous variances. For non-normal distribution, Mann–Whitney U test (two groups) and Kruskal–Wallis test followed by Dunn’s *post hoc* test (≥ three groups) were used. Statistical significance was set at *p* value <.05 was considered statistically significant.

## 3. Results

### 3.1. Decreased renal function and abnormal metabolism in DKD model mouse

**Figure 1.**
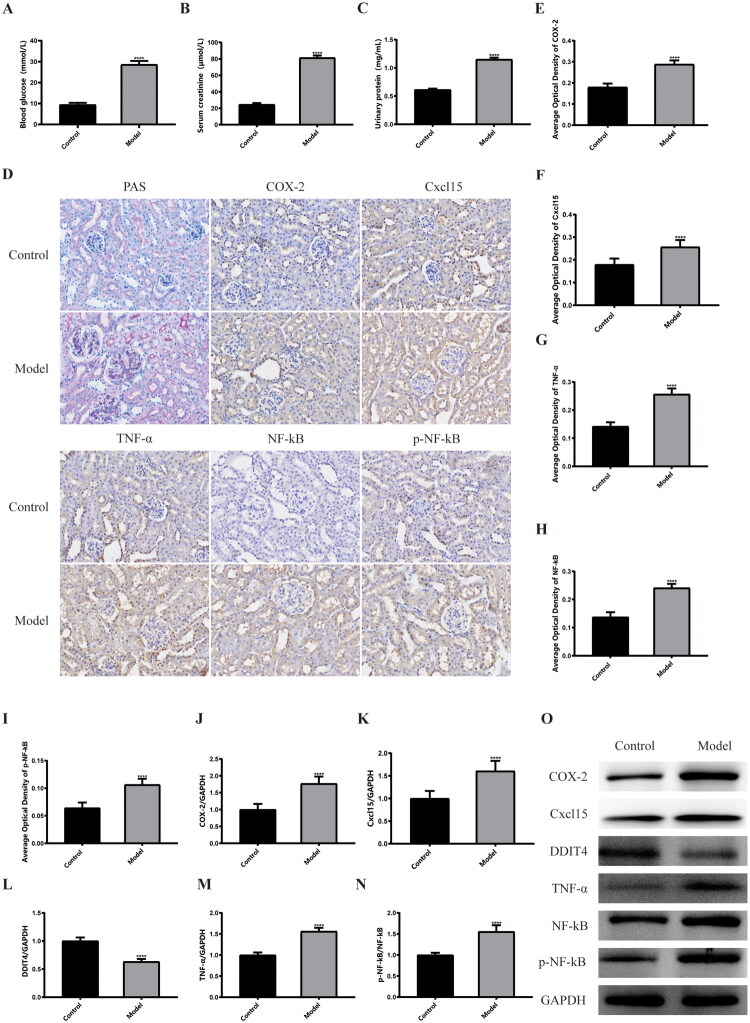
Biochemical indices, histopathology, and inflammation levels in a mouse model of diabetic kidney disease. (A) Blood glucose results of mouse. (B) Serum creatinine results of mouse. (C) Urinary protein results of mouse. (D) PAS staining and IHC staining results of mouse kidney samples (20X). (E) The average optical density of COX-2. (F) The average optical density of Cxcl15. (G) The average optical density of TNF-α. (H) The average optical density of NF-κB. (I) The average optical density of p-NF-κB. (J) The relative protein expression of COX-2 to GAPDH. (K) The relative protein expression of Cxcl15 to GAPDH. (L) The relative protein expression of DDIT4 to GAPDH. (M) The relative protein expression of TNF-α to GAPDH. (N) The relative protein expression of p-NF-κB to NF-κB. (O) Western blots of protein expression in mouse kidney samples as indicated. ****p < .001, compared with control group. GAPDH: glyceraldehyde-3-phosphate dehydrogenase; COX2: cyclooxygenase-2; Cxcl15: C-X-C motif chemokine ligand 5; DDIT4: DNA damage inducible transcript 4; TNF-α: tumor necrosis factor-α; NF-κB: nuclear factor-κB; PAS: Periodic acid Schiff.

At the animal level, biochemical markers were used to detect DKD progression in db/db mice. As shown in Figure 1(A–C), blood glucose (28.63 ± 1.71 mmol/l vs. 9.4 ± 0.87 mmol/l, *p* < .0001) and serum creatinine (24.56 ± 1.83 μmol/L vs. 81.37 ± 2.73 μmol/L, *p* < .0001), urinary protein (1.15 ± 0.03 mg/ml vs. 0.61 ± 0.02 mg/ml, *p* < .0001) were significantly higher than normal control mouse. At the same time, in performed to observe the content and distribution of glycogen in kidney tissue. The results showed that in the kidney tissues of the model group, the glomeruli showed ‘lobulated’ pathological changes, and more red or purplish-red cytoplasm was observed in the tissues, indicating more glycogen deposition. These results highlight the effectiveness of building a DKD model using db/db mice.

### 3.2. Changes in the expression of renal inflammatory factors and DDIT4 molecules in DKD model mouse

To study the changes in kidney inflammatory factors and DDIT4 molecules in DKD, we performed immunohistochemical staining and WB experiments on kidney samples. The results of IHC staining showed that the staining of inflammatory factors such as COX2, Cxcl15, TNF-α, NF-кB, and p-NF-кB in the kidney tissues of the DKD model group was mostly dark brown, whereas those of the control group were mostly negative or light yellow, indicating that the expression of inflammatory factors in the model group was significantly higher than that in the control group. At the same time, increased protein deposition was observed in the renal tubules of the model group, which was also consistent with the pathological changes observed in DKD. (Figure 1(D–1)). WB results also showed that the expression of inflammatory cytokines in the model group was significantly higher than that in the control group. Notably, the expression of DDIT4 in the model group was significantly lower than that in the control group (Figure 1(J–O)), suggesting that the expression of DDIT4 was inhibited in diabetic nephropathy, which was consistent with our previous findings. These results indicate that DDIT4 expression is inhibited and inflammatory factors are elevated during the development of DKD, which promotes pathological damage due to kidney inflammation. Combined with the results of previous studies, we believe that regulation of DDIT4 expression may be related to the progression of inflammation in DKD.

### 3.3. Renal macrophage infiltration and podocyte injury in DKD model mouse

**Figure 2.**
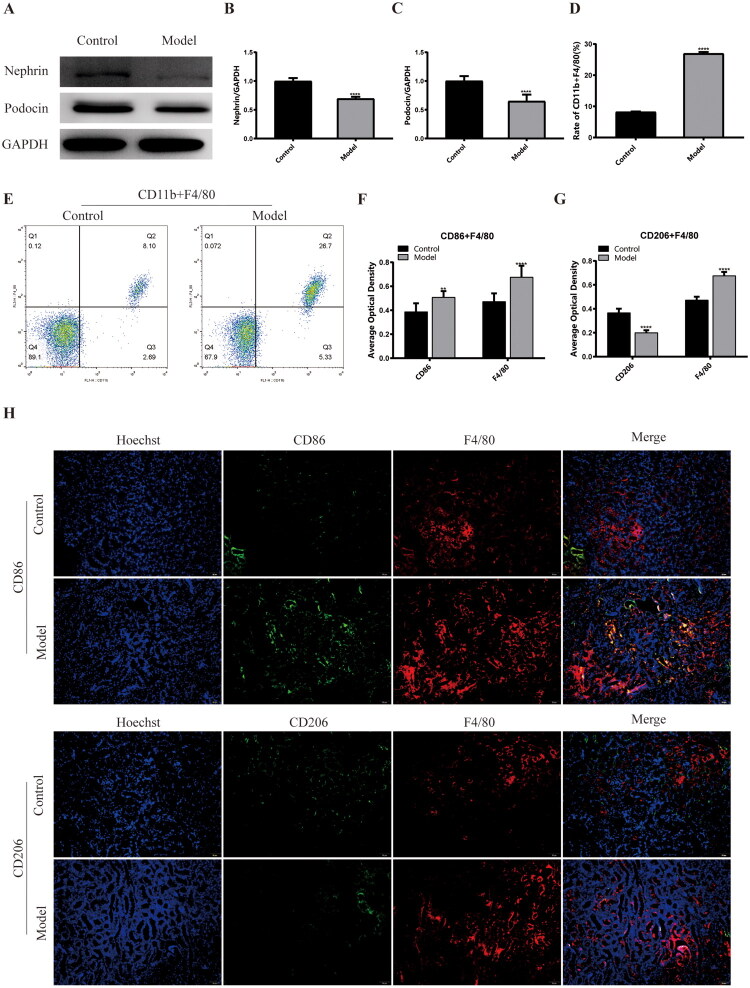
Expression of podocytes damage-related proteins and proportion of macrophage infiltration and polarization phenotype in the kidney of a mouse model of diabetic kidney disease. (A) Western blots of protein expression in mouse kidney samples as indicated. (B) The relative protein expression of nephrin to GAPDH. (C) The relative protein expression of podocin to GAPDH. (D) The rate of CD11b + F4/80 in macrophages in mouse kidney samples. (E) Detection of macrophage marker CD11b + F4/80 in mouse kidney tissue by cytometry. (F) The average optical density of CD86 and F4/80. (G) The average optical density of CD206 and F4/80. (H) Laser confocal results of CD86, CD206, F4/80 in mouse kidney samples. **p<.01, ****p<.001, compared with Control group.

Macrophages and podocytes were isolated from the mouse kidney tissues. The expression of Nephrin and Podocin proteins in podocytes was detected by WB experiment in podocytes. Nephrin and podocin are podocyte-specific proteins involved in the normal development and function of the glomerular filtration barrier. The results showed that the expression of nephrin and podocin proteins in the model group was significantly lower than that in the control group. Podocyte injury (Figure 2(A–C)) was observed in the DKD model. Next, flow cytometry quantitative detection of macrophages was performed, and the results showed that the proportion of macrophage marker CD11b + F4/80 (26.93 ± 0.49% vs. 8.22 ± 0.13%, *p* < .0001) in the model group was significantly higher than that in the control group. This suggests that there was increased macrophage infiltration in the kidneys of the DKD model (Figure 2(D,E)). We used immunofluorescence to detect the expression of M1-type macrophage marker CD86 and M2-type macrophage marker CD206 in these macrophages to further explore the polarization phenotype of these macrophages. We found that the expression of CD86 (0.51 ± 0.05 vs. 0.39 ± 0.07, *p* < .01) was significantly increased in the model group, but the expression of CD206 (0.2 ± 0.02 vs. 0.37 ± 0.03, *p* < .0001) was significantly decreased compared with the control group. The results showed that in the DKD model, a large number of macrophages infiltrated by the kidneys were mainly M1-type macrophages (Figure 2(F– H)). Combined with previous research results, we believe that in the progression of DKD, a large number of M1-type macrophages infiltrated the kidney tissue, and the expression of inflammatory factors increased, which induced the rapid development of inflammation, leading to the structural destruction of kidney tissue and cells and the dysfunction of physiological function.

### 3.4. Construction and phenotypic verification of activated macrophage model

**Figure 3.**
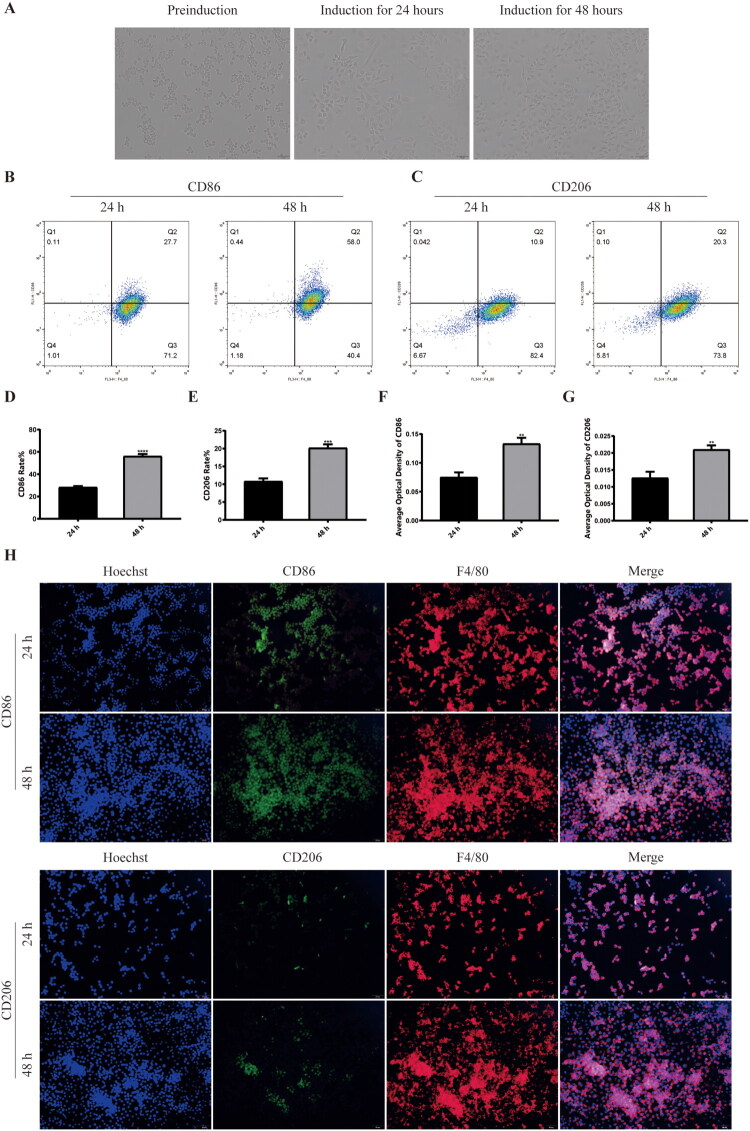
Induction and characterization of M1-type macrophages. (A) Cultured Raw264.7 cells with PMA and LPS for 24h and 48h for induction of polarized macrophages. (B) Cytometry flow results of CD86 after induction for 24 h and 48 h. (C) Cytometry flow results of CD206 after induction for 24 h and 48 h. (D) The CD86 rate after 24 and 48 h of induction. (E) The CD206 rate after 24 and 48 h of induction. (F) The average optical density of CD86 after induction for 24 h and 48 h. (G) The average optical density of CD206 after induction for 24 h and 48 h. (H) Laser confocal results of CD86 and CD206 after induction for 24 h and 48 h. **p < .01, ***p < .005, ****p < .001, compared with control group.

At the cellular level, Raw264.7 cells were induced to M1 polarization phenotype macrophages using phorbol 12-myristate 13-acetate (PMA) and lipopolysaccharide (LPS). Cells were induced for 24 h and 48 h, respectively. Microscopic observations showed that before induction, the cells were full in shape, uniform in size, and had good refractive properties. After induction, the cell shape gradually became irregular, the cell body enlarged, and the cell surface extended to pseudopodia, indicating that activated macrophages (Figure 3(A)) were successfully constructed. We then performed flow cytometry and found that 48 h after induction, compared to 24 h, both CD86 (56.17 ± 1.85% vs. 28.33 ± 1.01%, *p* < .0001) and CD206 (20.17 ± 1.01% vs. 10.82 ± 0.83%, *p* < .001) were significantly increased, and CD86 was the most significant. This indicates that we successfully constructed an activated M1 macrophage model, with the best results at 48 h of induction (Figure 3(B–E)). We then performed immunofluorescence tests, and the results showed that the fluorescence density of CD86 (0.13 ± 0.01 vs. 0.08 ± 0.01%, *p* < .01) was significantly increased in the cell model 48 h after induction, which was consistent with previous results and strengthened the reliability of the results (Figure 3(F–H)). In the follow-up experiment, we induced Raw264.7cells for 48 h to construct the M1 polarized macrophage model.

### 3.5. DDIT4 plasmid transfection efficiency visualization

**Figure 4.**
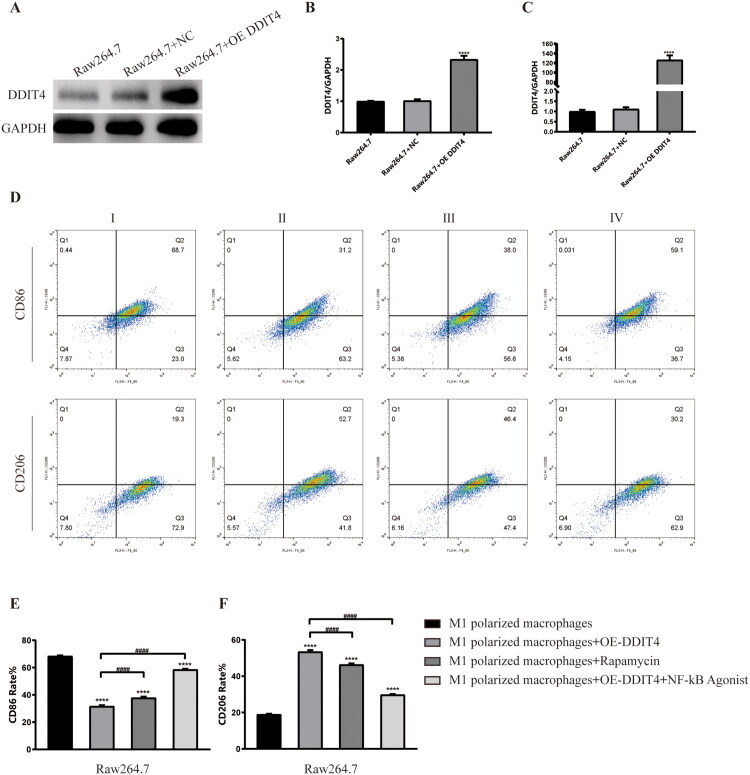
Transfection of DDIT4 and its role in the polarization phenotype of macrophages. (A)Western blots of protein expression in Raw264.7 cells after transfection DDIT4. (B)The relative protein expression of DDIT4 to GAPDH. (C) qRT-PCR results of Raw264.7 cells after transfection DDIT4. (D) The expression of CD86 and CD206 in M1-type macrophages was detected by flow cytometry sorting after different intervention conditions. (E) The rate of CD86 expression was detected by flow cytometry. (F) The rate of CD206 expression was detected by flow cytometry. NC: negative control; OE: overexpression; GAPDH: glyceraldehyde-3-phosphate; DDIT4: DNA damage inducible transcript 4; I:M1 polarized macrophages; II: M1 polarized macrophages + OE-DDIT4; III: M1 polarized macrophages + rapamycin; IV:M1 polarized macrophages + OE-DDIT4 + NF-kB Agonist. ****p<.001, compared with group I. ###*p*<.005, ####*p*<.001, compared with group II.

To study the regulatory effect of DDIT4 on the macrophage model, we used the DDIT4 plasmid for cell transfection experiments, and performed WB and RT-PCR assays to determine transfection efficiency. The results showed that there was no significant change in DDIT4 expression after transfection with the empty plasmid compared with the blank control group, and DDIT4 gene expression was significantly up-regulated after transfection with the DDIT4 overexpression plasmid (Figure 4(A–C)). These results indicate that the DDIT4 plasmid was successfully delivered into the cells and expressed.

### 3.6. DDIT4 down-regulation of mTOR level promotes phenotypic transformation of macrophages and inhibition of inflammatory factors

**Figure 5.**
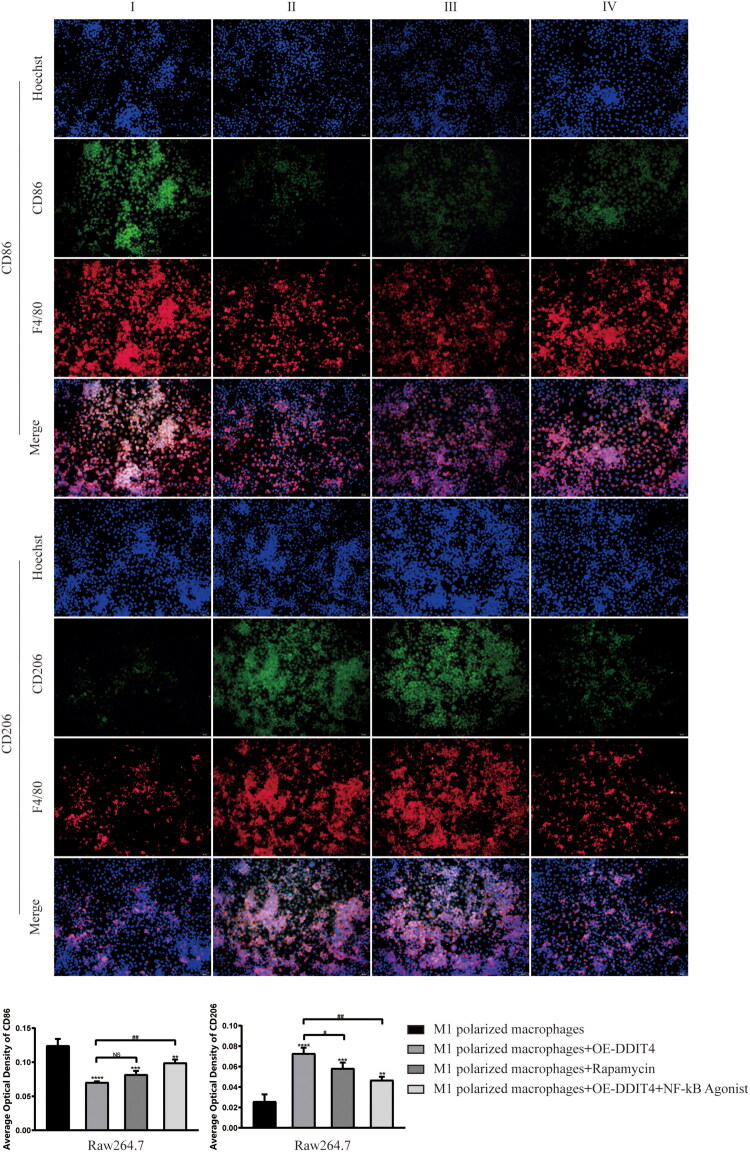
Immunofluorescence detection of CD86 and CD206 verified the role of DDIT4 in the polarization phenotype of macrophages. I: M1 polarized macrophages; II: M1 polarized macrophages + OE-DDIT4; III: M1 polarized macrophages + rapamycin; IV:M1 polarized macrophages + OE-DDIT4 + NF-kB agonist. **p < .01, ***p < .005, ****p < .001, compared with group I. NS: *p* ≥ .05, #*p* < .05, ##*p* < .01, compared with group II.

**Figure 6.**
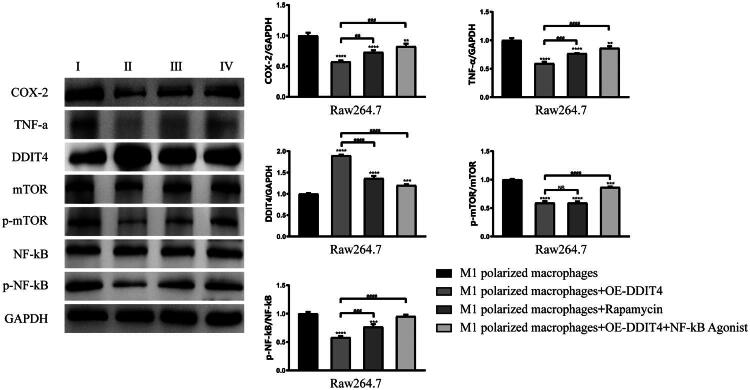
Western Blotting test detected DDIT4-mTOR pathway and expression of inflammatory factors after different intervention conditions. I: M1 polarized macrophages; II: M1 polarized macrophages + OE-DDIT4; III: M1 polarized macrophages + rapamycin; IV:M1 polarized macrophages + OE-DDIT4 + NF-kB agonist. **p < .01, ***p < .005, ****p < .001, compared with group I. NS: *p* ≥ .05, ##*p* < .01, ###*p* < .005, ####*p* < .001, compared with group II. COX2: cyclooxygenase-2; TNF-α: tumor necrosis factor-α; DDIT4: DNA damage inducible transcript 4; mTOR: The mammalian target of rapamycin; NF-κB: nuclear factor-κB; GAPDH: glyceraldehyde-3-phosphate dehydrogenase.

To verify the regulation of the DDIT4-mTOR pathway on phenotypic transformation and inflammation of macrophages, activated M1-type macrophages were divided into four groups for experiments, I: M1-polarized macrophages, II: M1-polarized macrophages + overexpressing DDIT4, III: M1-polarized macrophages + rapamycin (mTOR inhibitor), and IV: M1-polarized macrophages + overexpressed DDIT4 + NF-кB agonists. Flow cytometry results showed that CD86 (31.7 ± 0.7% vs. 68.43 ± 0.46%, *p* < .0001) expression in M1-polarized macrophages was down-regulated after DDIT4 overexpression. The expression of CD206 (53.47 ± 0.93% vs. 18.97 ± 0.35%, *p* < .0001) was up-regulated. The same result was observed after the addition of rapamycin; however, the effect was not as significant as that of DDIT4 overexpression. When the NF-кB agonist was added to enhance the activation of the inflammatory pathway, the regulatory effect of DDIT4 overexpression on the changes in CD86 and CD206 contents in cells was weakened. However, compared to simple M1-polarized macrophages, CD86 (58.57 ± 0.5% vs. 68.43 ± 0.46%, *p* < .0001) and CD206 (29.83 ± 0.32% vs. 18.97 ± 0.35%, *p* < .0001) still showed different expression changes (Figure 4(D–F)). The same result was observed in the immunofluorescence experiment (Figure 5), which strengthened the accuracy of the experimental results. WB experiment results showed that compared with M1polarized macrophages, after overexpression of DDIT4 or addition of rapamycin, the expression of COX-2, p-mTOR, p-NF-кB, and TNF-α in the cells was down-regulated, while the expression of DDIT4 was up-regulated, and the overexpression of DDIT4 produced the most significant effect. After overexpression of DDIT4 and addition of NF-KB agonists at the same time, the inflammatory pathway effect was strengthened. We observed that the expression of inflammatory factors and the activation of mTOR showed an increasing trend, but it was still lower than that of simple M1-type macrophages. These results indicate that overexpression of DDIT4 can promote the transformation of macrophages to the M2 type and inhibit the expression of inflammatory factors, and this effect is related to the regulation of mTOR expression. Our experimental results also emphasized that the overexpression of DDIT4 had a more positive therapeutic effect than the application of mTOR inhibitors, and DDIT4 still had a considerable therapeutic effect under the condition of enhanced inflammatory effects (Figure 6).

### 3.7. DDIT4 regulates macrophages and inflammation to reduce podocyte damage

**Figure 7.**
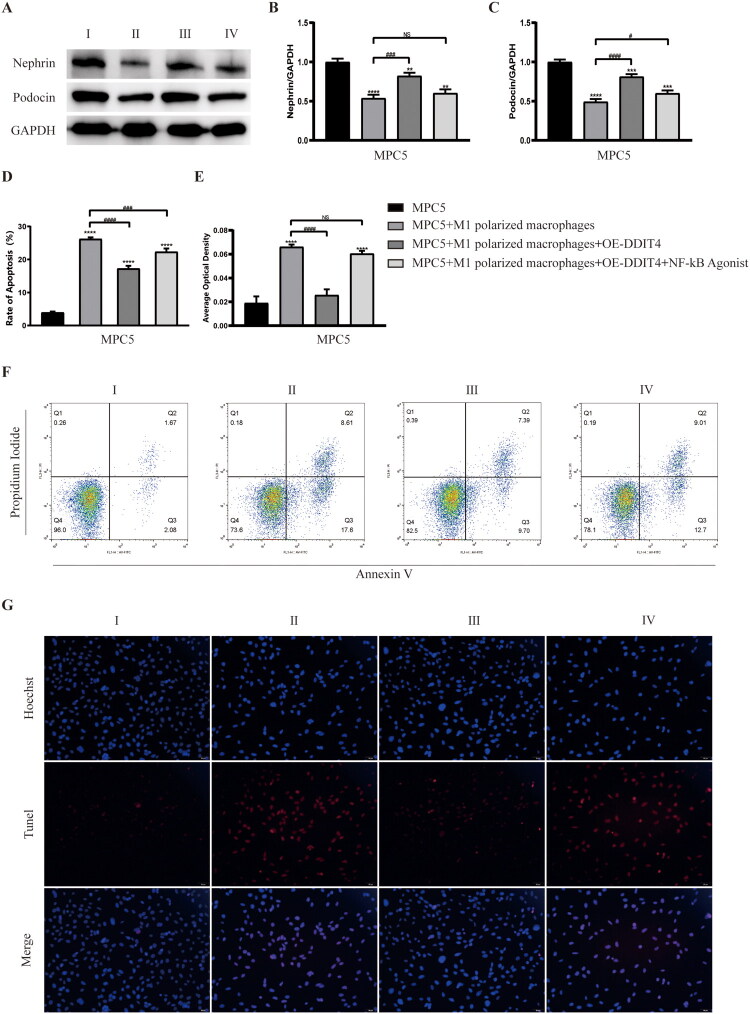
Verification of the protective effect of DDIT4 on podocyte injury. (A) Western blots of protein expression after different intervention conditions as indicated. (B) The relative protein expression of nephrin to GAPDH. (C) The relative protein expression of podocin to GAPDH. (D) The apoptosis rate after different intervention conditions as indicated. (E) The average optical density of apoptosis after different intervention conditions as indicated. (F) Results of flow cytometry. (G) Results of Tunel staining. MPC5: Mouse Podocyte Clone-5; I: MPC5 + control medium; II: MPC5 + medium from the group of M1 polarized macrophages; III: MPC5 + medium from the group of M1 polarized macrophages + OE-DDIT4; IV: MPC5 + medium from the group of M1 polarized macrophages + OE-DDIT4 + NF-κB agonist. **p < .01, ***p < .005, ****p < .001, compared with group I. ###*p* < .005, ####*p* < .001, compared with group II.

**Figure 8.**
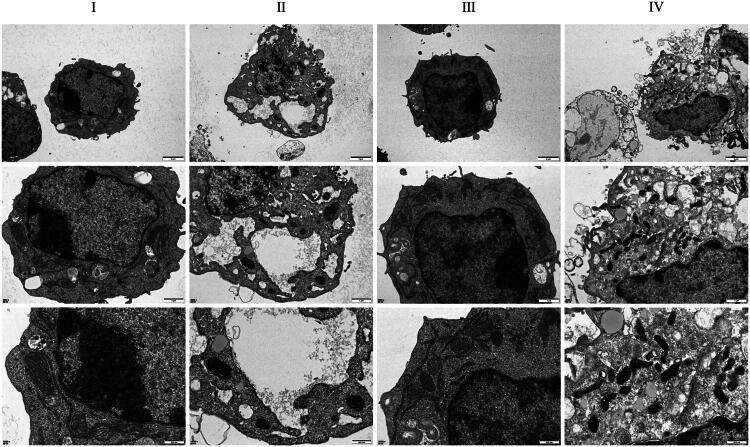
Transmission electron microscope observation results of MPC5 cells. Each group, from top to bottom, is 2,500X, 5,000X, 10,000X with the same field of vision. I: MPC5 + control medium, II: MPC5 + medium from the group of M1 polarized macrophages, III: MPC5 + medium from the group of M1 polarized macrophages + OE-DDIT4, IV: MPC5 + medium from the group of M1 polarized macrophages + OE-DDIT4 + NF-κB agonist.

Next, we retained the media after group modeling and intervention in Step 6, cultured podocytes, and used normal media culture as a control to observe podocyte injury. WB blot results showed that compared with the normal culture medium group, the expression levels of nephrin and podocin in podocin the M1-polarized macrophages group were down-regulated, and this phenomenon could be improved after culture in the DDIT4 high expression group. When the overexpression of DDIT4 was combined with the addition of the NF-кB agonist, the expression levels of nephrin and podocin proteins in podocin cells were not significantly different from those in the M1-polarized macrophage medium group (Figure 7(A–C)). In the subsequent flow cytometry and TUNEL staining experiments, we observed that compared with the normal medium group, the apoptosis rate of podocytes cultured in the M1-polarized macrophage group was significantly up-regulated, and this phenomenon could be improved after culture in the DDIT4 high expression group. Under conditions of DDIT4 overexpression, the apoptosis rate of podocytes increased after the addition of the NF-кB agonist, which was not significantly different from that of M1-polarized macrophages (Figure 7(D–G)). The results of transmission electron microscopy showed that compared with the normal medium group, the damage degree of podocytes cultured in the M1-polarized macrophages group was significantly higher than that in the normal medium group, and this phenomenon could be improved after culture in the DDIT4 high expression group. When the NF-κB agonist was added at the same time, the cultured podocytes again showed significant damage (Figure 8). These results suggest that overexpression of DDIT4 inhibits the progression of DKD inflammation by regulating the polarization phenotype of macrophages and plays a protective role in podocytes.

## 4. Discussion

**Figure 9.**
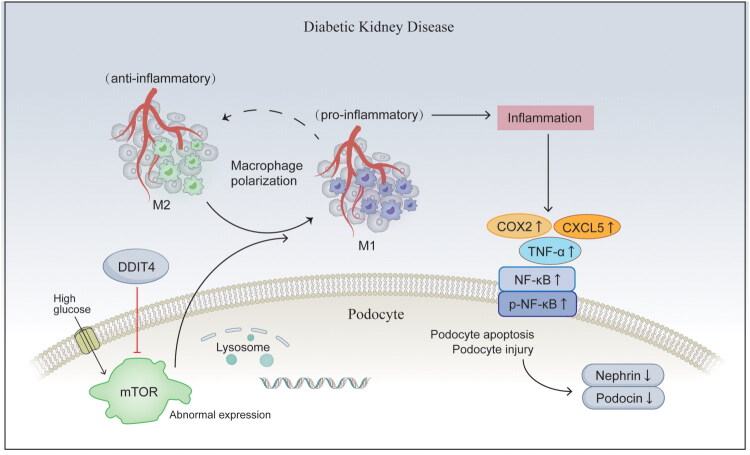
The regulatory mechanism of DDIT4-mTOR signaling pathway in the development of DKD. In high-glucose environment, the abnormal expression of mTOR mediates the transformation of macrophage phenotype, promotes inflammation, induces the up-regulation of COX2, CXCL15, TNF-α, NF-κB and p-NF-κB, thereby damaging podocytes, inhibiting the expression of podocin and nephrin, and aggravating the progression of DKD. Increasing DDIT4 expression can help to reverse the damage mechanism and protect podocytes. DDIT4: DNA damage inducible transcript 4; mTOR: The mammalian target of rapamycin; COX2: cyclooxygenase-2; Cxcl15: C-X-C motif chemokine ligand 5; TNF-α: tumor necrosis factor-α; NF-κB: nuclear factor-κB.

In recent years, with the increasing prevalence of DM, the number of patients with DKD has increased [23]. The occurrence and development of DKD are involved in a variety of pathophysiological disorders, including hemodynamic abnormalities, oxidative stress, cell death, and epigenetic regulation [24,25]. Currently, the clinical treatment of DKD mainly includes angiotensin-converting enzyme inhibitors (ACEIs), angiotensin II receptor antagonists (ARBs), and sodium-dependent glucose transporter 2 (SGLT2) inhibitors [26,27]. However, the role of these treatments in slowing the progression of DKD remains unclear. Based on the complex pathogenesis and unsatisfactory therapeutic effects of DKD, it is necessary to study its pathogenesis and formulate appropriate therapeutic methods. A large body of evidence suggests that inflammation plays an important role in the progression of DKD [4,28,29]. A high-glycemic environment induces abnormal regulation of inflammatory factors and cytokines and related signaling pathways in damaged glomeruli and renal tubules, including TNF-α, CC chemokine 2 (CCL2), also known as monocyte chemoattractant protein-1 (MCP-1), NF-кB, AKT/mTOR signaling pathway, TGF-β/Smad signaling pathway, etc. [30,31]. Through the action of various molecules and pathways, cell damage is induced, the kidney filtration barrier is destroyed, kidney damage is aggravated [4,32], and the development of DKD is promoted [10]. We established an animal model of DKD using db/db mice. The levels of urinary microalbumin and blood creatinine were consistent with the expected changes in the DKD biochemical indices. Kidney tissue samples were stained with PAS, and it was observed that the positive expression of glycogen increased in the model group and was significantly different from that in the control group. WB and immunohistochemical staining analysis showed that the levels of the inflammatory factors COX2, CXCL15, TNF-α, NF-κB and p − NF − κB in the model group were significantly higher than those in the control group, indicating that DKD-related renal pathological injury was closely related to inflammation, which was consistent with the results of previous studies. COX2 is an enzyme that is induced to be expressed during inflammation. It is also a key downstream inflammatory mediator of the mTOR/NF-κB pathway. In kidney inflammation, the upregulation of COX2 can increase the production of prostaglandins, thus promoting the inflammatory response and pain sensitivity. NF-κB is a transcription factor that controls the expression of a variety of inflammatory genes [33,34]. When stimulated by inflammatory signals, NF-κB is activated and transferred to the nucleus, promoting the expression of inflammatory genes. p-NF-кB is the activated forms of NF-кB. Phosphorylated NF-кB has a higher transcriptional activity and can promote the expression of inflammatory genes more effectively. TNF-α is a strong cytokine that promotes the development of inflammation, activates various cell types, promotes the production of inflammatory cytokines, and enhances the function of immune cells. Previous studies have demonstrated that TNF-α is a hallmark cytokine of M1 macrophages and directly induces podocyte apoptosis [35,36]. CXCL15 is a CXC chemokine with neutrophil chemotactic activity. The biological function of CXCL15 is most similar to that of human CXCL8, which can lead to the activation of NF- кB and AP-1 complexes through downstream signaling pathways. It is also considered a key chemokine for macrophage infiltration in DKD [37–39]. During the development of kidney inflammation, TNF-α can activate the NF-кB pathway, enhance the expression of COX2 and other inflammatory genes, induce the production of more inflammatory mediators, and further amplify the inflammatory response. Therefore, these factors interact with kidney inflammation and jointly promote the development and maintenance of inflammatory processes [40,41]. Macrophages play important regulatory roles in the occurrence and development of inflammation. Studies have confirmed that in the early stage of DKD, macrophages migrate to the kidney in large numbers, forming immune infiltration and releasing inflammatory mediators, aggravating kidney injury and leading to chronic inflammation [42–44]. Macrophage-mediated podocyte apoptosis is considered an important factor in the development of DKD [16]. Therefore, effective inhibition of macrophage-mediated inflammatory damage could be a research direction for the treatment of DKD. Podocytes and macrophages were isolated from the kidney tissues of the animal model. WB results showed that the expression of nephrin and podocin proteins in the kidney tissues of the DKD model group was significantly lower than that in the normal group. Cytometry and immunofluorescence tests showed that the kidneys of the DKD model group had a higher infiltration of macrophages. M1-type macrophages, which promote inflammation, were dominant. Macrophages can participate in the occurrence and development of DKD by inducing inflammation and damaging podocytes. A recent study has shown that the increased expression of DDIT4 can down-regulate of the expression levels of pro-inflammatory cytokines such as NF-κB and oxidative stress-related markers by regulating macrophages [21]. Our previous studies revealed that DDIT4 can play a role in enhancing autophagy and alleviating oxidative stress by regulating the mTOR pathway, the target protein of mammalian rapamycin, and alleviating the occurrence and development of DKD [45,46]. mTOR is a serine/threonine protein kinase belonging to the phosphatidylinositol-associated protein kinase family, which regulates cell growth, metabolism, proliferation, and survival, and plays an important role in the signaling pathway network related to kidney disease [47,48]. Studies have found that mTOR can promote the progression of DKD by regulating autophagy, fibrosis, oxidative stress, and inflammation, etc. [49,50] It has also been demonstrated that mTOR plays an important role in regulating the phenotype polarization of macrophages toward M1 and M2 [51]. Macrophage activation syndrome (MAS), is a life-threatening systemic inflammatory disorder. T lymphocytes and well-differentiated macrophages are over-activated and proliferate, thus producing a large number of cytokines, resulting in an abnormal immune state of the body [52]. IL-1, a cytokine that activates MAS, induces mTORC1 activation. Activated mTORC1 induces the expansion of inflammatory monocytes and promotes inflammatory responses. Application of the mTOR inhibitor rapamycin can reduce the systemic inflammatory response [53]. Macrophages activate and polarize into functionally distinct subpopulations, a process associated with extensive epigenetic modifications, transcriptional reprogramming, and metabolic changes. Activated macrophages can be stimulated by various environmental factors to differentiate into M1-type macrophages and M2-type macrophages. M1-type macrophages have anti-angiogenic effects and promote chronic inflammation. M2-type macrophages can fight inflammation and promote tissue repair [54]. Previous studies have suggested that M1-type macrophage polarization is related to glycolysis, the pentose phosphate pathway, and fatty acid synthesis, and mTORC1 activation can enhance the cellular glycolysis process, which is considered a feature of pro-inflammatory immune cells [55,56]. It has also been suggested that upstream activators of mTOR, such as ketamine or endothelial growth factor (EGF), can enhance the polarization of macrophages toward inflammatory phenotypes [57,58]. In addition, inhibition of mTOR can reduce the expression of MCP-1 [59]. MCP-1 is a key chemokine that regulates the migration and infiltration of macrophages. Diabetes can induce the production of renal MCP-1, renal macrophage infiltration, and promote the occurrence and development of DKD. When the expression of MCP1 is reduced in the cellular environment, the number of macrophages at the inflammatory site can be reduced and disease progression can be alleviated [60]. In conclusion, the proinflammatory effect of macrophages through the regulation of the mTOR pathway is a worthy direction to explore in the study of DKD. At the same time, DDIT4, a recognized endogenous inhibitor of mTOR [61], has been confirmed to have a protective effect on the kidney in the diabetic environment. In this study, we conducted WB experiments on kidney tissues of DKD animal models, and again verified that DDIT4 expression in kidney tissues of the DKD model group was significantly lower than that of the control group. It was also pointed out in a clinical study that serum DDIT4 levels in hyperlipidemia patients were significantly reduced, and the serum DDIT4 content was negatively correlated with blood glucose, blood lipid, insulin resistance, and inflammatory markers, which is consistent with our research conclusion [62]. Subsequently, we further explored the regulatory mechanism of DDIT4/mTOR involvement in macrophage polarization, and verified whether this mechanism can play an alleviating role in the development of DKD. RAW264.7 were induced to become M1-polarized macrophages using PMA and LPS, and the macrophage markers CD86 and CD206 were significantly elevated 48 h after induction by immunofluorescence and cytometry. Next, we performed a DDIT4 plasmid transfection experiment, and confirmed the successful transfection of the plasmid by RT-PCR and WB experiments. Subsequently, we verified the role of DDIT4 in macrophage regulation. Using immunofluorescence and flow cytometry, we found that after overexpression of DDIT4, the expression of CD86 in cells was down-regulated, and the expression of CD206 was upregulated. CD86 is a co-stimulatory molecule expressed on the surface of immune cells, including macrophages, dendritic cells, and B lymphocytes. It is often considered a marker for M1-type macrophages [63]. CD206, also known as mannose receptor type C-1 (MRC1), is a cell surface protein that is abundant in macrophages and dendritic cells and is usually expressed in M1-type macrophages [64]. Similar results were observed in the group treated with rapamycin; however, the effect was not significant compared with the overexpression of DDIT4. The expression levels of CD86 and CD206 were similar to those of M1-polarized macrophages when DDIT4 overexpression was combined with NF-кB agonists, but there were still significant differences. These results suggest that DDIT4 can promote the transformation of macrophages into anti-inflammatory M2-type macrophages, and may play a role in inhibiting the expression of mTOR. Nuclear factor-κB (NF-κB) is a transcription factor that regulates the expression of pro-inflammatory cytokine genes. The NF-κB signaling pathway is inhibited by the IκB protein complex, including inhibitor of κB-α (IκBα) and inhibitory κB kinase-β (IKKβ) [11]. Phosphorylation of IKKβ subsequently leads to the phosphorylation of IκBα, thereby activating the NF-κB signaling pathway [65,66]. Studies have indicated that mTORC1 binds to and phosphorylates IKKα and IKKβ, thereby enhancing their kinase activity. IKKα/IKKβ then phosphorylates IκB, inducing the release and activation of NF-κB, which promotes the initiation and progression of inflammation [67]. Next, we detected the expression levels of mTOR and inflammatory factors by WB blotting. The results showed that the expression levels of p − mTOR/mTOR and p-NF-кB/NF-кB, inflammatory factors COX-2, and TNF-α were down-regulated in cells overexpressing DDIT4, and the results were more significant than those in the rapamycin group. This suggests that DDIT4 can regulate the transformation of macrophages from the M1 to M2 type and reduce the release of inflammatory factors by inhibiting the activation of mTOR and NF-κB. Combined with previous experiments, we further explored whether this regulatory effect of DDIT4 could alleviate cell damage caused by inflammation in DKD. Subsequently, we cultured podocytes in the media of the above groups for 24 h and used normal media as the control. Western results of WB showed that the expression of nephrin and podocin proteins in podocin cultured in the overexpressed DDIT4 culture medium group was significantly higher than that in the M1-polarized macrophage culture medium group. The results of flow cytometry and tunnel staining showed that the proportion of podocyte apoptosis in the M1-polarized macrophage culture medium group was significantly higher than that in the control group, and the proportion of podocyte apoptosis was significantly reduced when cultured in the culture medium of the overexpressed DDIT4 group. We also found that the apoptosis rate of podocytes was up-regulated in the medium with DDIT4 overexpression and NF-κB agonist, but there was still a significant downward trend of apoptosis compared with the medium with M1 polarized macrophages alone. This suggests that DDIT4 can effectively protect podocytes by influencing the macrophage phenotype and inhibiting the activation of inflammatory factors (Figure 9).

It is noteworthy that although this study, by exploring the regulatory role of DDIT4 in macrophages, has opened up a promising therapeutic avenue for treating DKD, it still has certain limitations. These include the lack of validation of DDIT4’s regulatory effects using human primary macrophages, the need for further analysis of the phosphorylation status of mTOR downstream effector molecules (such as S6K/4E-BP1), and the fact that the conditioned medium experiments did not fully replicate the microenvironment of direct macrophage-podocyte contact. In future research, we will combine single-cell sequencing and organoid co-culture models to deepen the exploration of the underlying mechanisms.

## 5. Conclusion

In conclusion, exploring the regulatory effect of DDIT4 on macrophages opens a promising therapeutic pathway for the treatment of DKD. Changes in mTOR and inflammatory factors have been observed *in vivo* and *in vitro*, highlighting their significance in the pathogenesis of DKD. The ability of DDIT4 to mediate transformation of the macrophage phenotype and reduce inflammation reveals its potential as a new drug discovery target for DKD. These findings open new avenues for innovative drug discovery approaches for chronic kidney disease treatment. Further exploration and validation of the therapeutic potential of DDIT4 may provide effective interventions to address the unaddressed clinical needs in the treatment of DKD.

## Supplementary Material

Supplementary Material 2

Supplementary Material 1

## Data Availability

Data will be made available on request.
